# Magnifying the importance of collecting race, ethnicity, industry, and occupation data during the COVID-19 pandemic

**DOI:** 10.4178/epih.e2021095

**Published:** 2021-11-06

**Authors:** Sai Krishna Gudi, Sophia M. George, Komal Krishna Tiwari

**Affiliations:** 1 College of Pharmacy, Rady Faculty of Health Sciences, University of Manitoba, Winnipeg, MB, Canada; 2 Information Management & Analytics, Epidemiology and Surveillance, Health, Seniors and Active Living, Winnipeg, MB, Canada; 3 Department of Pharmacy Practice, NGSM Institute of Pharmaceutical Sciences, NITTE University (Deemed), Mangalore, India; 4 Department of Occupational Therapy, College of Rehabilitation Sciences, University of Manitoba, Winnipeg, MB, Canada

**Keywords:** Race, Ethnicity, Industry, Occupation, COVID-19

## Abstract

The contagiousness of coronavirus disease-2019 (COVID-19) led to the imposition of historical lockdowns in various countries. No scientific mind could have made accurate projections of the tremendous impact that COVID-19 would have on nations, communities, and the global-wide economy. Meanwhile, millions of workers have lost their jobs, while healthcare workers are overwhelmed and are reaching a state of mental and physical exhaustion. With the uncontrollable spread, researchers have been working to identify factors associated with COVID-19. In this regard, race, ethnicity, industry, and occupation have been found to be predominant factors of interest. However, unfortunately, the unavailability of such information has been a difficult reality. Since race, ethnicity, and employment are essential social determinants of health and could serve as potential risk-factors for COVID-19, collecting such information may offer important context for prioritising vulnerable groups. Thus, this perspective aims to highlight the importance and need for collecting race, ethnicity, and occupation-related data to track and treat the racial/ethnic groups that have been most strongly affected by the COVID-19 pandemic. Collecting such data will provide valuable insights and help public health officials recognise workplace-related outbreaks and evaluate the odds of various ethnic groups and professions contracting COVID-19.

## INTRODUCTION

Scientific research revolving around the coronavirus disease 2019 (COVID-19) pandemic has yielded preliminary evidence of the higher vulnerability of certain races, socioeconomic groups, and occupations [1]. The racial disparities that have long existed around the world have only become exaggerated during the pandemic. Scientific reports from the Centres for Disease Control and Prevention (CDC) suggest that the Black community, which encompasses around 13% of the United States population (according to the United States Census Bureau, 2018), accounted for almost 30% of COVID-19 cases, where Latinos, who constitute 18% of the nation’s population, accounted for only 17% of registered COVID-19 cases [2]. This unequal distribution is also apparent in hospitalisation rates. According to 2020 data obtained from the Kaiser Family Foundation and the CDC, the highest percentages of mortality were also recorded amongst Blacks and Latinos [3]. With the pandemic surge in late 2020, the United States started to stratify data about COVID‐19 cases based on race and ethnicity, and there is emerging evidence that people of colour (POC), especially Black Americans, are at an increased risk for contracting, being hospitalised, and dying from COVID-19 [4].

Various reasons have been proposed to explain these disparities; for example, it has been pointed out that POC are likely to be more socioeconomically underprivileged, reside in highly dense populated areas, have more comorbid health conditions, and be employed in roles that cannot be performed remotely. Furthermore, other structural factors such as discrimination and racism make these groups vulnerable to COVID‐19 [5]. A vast majority of the Black community is predominantly employed in restaurants, retail, and hospitality settings, which are particularly at risk for loss of income during the pandemic. In short, members of the Black community are commonly engaged in jobs that cannot be done from home and use public transportation, which puts them at risk for exposure to COVID-19 [3]. Moreover, disparities and discrimination within the healthcare system may also contribute to worse outcomes within certain specific groups, occupations, and industries, particularly among those who work in healthcare and other essential services [6]. It was also found that Black, Asian, and Hispanic workers were more likely to be employed in the food processing and animal slaughtering industry and transportation (bus drivers and flight attendants), where frequent exposures to COVID-19 infection and significant outbreaks have taken place [7].

The World Health Organization (WHO) has stated that the ease of transmission is enhanced in close-contact settings, crowded places, and enclosed spaces with poor ventilation [8]. Keeping that in mind, specific work settings and occupations are predisposed to involve a heightened risk of infections. As is well-known, certain occupations, such as healthcare workers (HCWs), those employed with jobs that mandate mass interactions, and those in the civil services, have been on the front-line since the outbreak of COVID-19 started [9]. They have been pitching in extra hours to manage the rampant increase in patient load and execute the orders issued by the administrative heads of every country. These services involve substantial sacrifices, as many of these workers were infected themselves and eventually lost their battle to the virus. The reasons for this disproportionate distribution of the virus amongst certain occupations slowly began to gain attention and precedence in the scientific community. As one of the most affected states in the United States, with more than 4 million cases and 64,000 fatalities as of late July 2021, the per-capita excess mortality in California is relatively high among Blacks and Latinos [10]. Investigations have hypothesised workplace settings as a risk factor for mortality; however, whether excess mortality varies across race, ethnicity, occupation, and industry has not been fully examined. Hence, collecting such information could point to opportunities for interventions among certain vulnerable groups facing heightened transmission risks.

## NEED FOR COLLECTING RACE, ETHNICITY, AND OCCUPATION DATA

According to the data reported by the CDC, a 33% morbidity rate has been identified among non-Hispanic Black individuals, whereas they comprise 18% of the total population. In contrast, a 45% morbidity rate has been identified among non-Hispanic Whites, who comprise 59% of the total population [4]. There has been a significant difference between these racial groups, wherein Black individuals have been disproportionately affected by COVID-19 in terms of hospitalisations compared to non-Hispanic Whites (Table 1). This is preliminary evidence of the fact that developed countries like the United States have begun realising the importance of collecting race, ethnicity, and occupation information after recognising the clear scientific correlation between these risk factors and the likelihood of contracting COVID-19. The accurate recognition and documentation of individuals’ occupational engagements and their racial information could prove very useful in identifying susceptible professions and populations, mitigating workplace breakouts, establishing safety measures, and facilitating in-depth research on the correlations of these factors with COVID-19 incidence.

The workplace is considered a possible venue for the transmission of infection, and various occupations face different risks for COVID‐19 exposure [12]. These occupation-acquired exposures may contribute to racial and ethnic disparities in COVID‐19 cases and fatalities [8]. Efforts to control the spread of COVID‐19 infection in the workplace can help protect workers, further reducing health disparities. Research conducted in different parts of the globe has undisputedly established that workers in certain occupations and industries are at a heightened risk of testing positive for COVID-19, specifically those who are part of the medical profession and those who work in industries that perform essential roles in the community. A study published by Zhang [13] in late 2020 focused on calculating the differential risk of contracting COVID-19, utilising the indicators obtained from the Occupational Information Network database, which were used to tally the total number of confirmed cases as published by the Washington State Department of Health. In accordance with a study conducted amongst 120,000 residents of the United Kingdom, individuals employed within healthcare settings were at a 7 times higher risk of being infected [14]. A survey performed in mid-2020 at a United Kingdom teaching hospital confirmed that the highest rates were found amongst COVID-19 front-line workers (21%). It is also interesting to note that the odds of contracting COVID-19 amongst social servants was 3 times higher than the common working groups [15].

Food processing plants in various nations have also been identified as hotspots for COVID-19 outbreaks, with reports of greater than 500 confirmed cases from a single site as stated by the Wellcome Open Research COVID-19 working group [16]. As per the European Centre for Disease Prevention and Control, around 1,376 clusters of COVID-19 outbreaks in occupational settings were identified all across Europe during the period between March and early July 2020 [17]. It is compelling to note that officials from Colorado working with the CDC also obtained results that agree with the findings of the United States, United Kingdom, and other European nations. They concluded that those employed in healthcare settings accounted for the highest percentage of positive tests (38%), followed by individuals working within office settings (17%). Public servants occupied third place, accounting for 7% of the infected, and lastly, personnel actively involved with manufacturing, including meatpacking, were responsible for 6% of the total COVID-19 positive cases [18]. These findings reiterate that a thorough investigation of occupational risk factors can mitigate workplace outbreaks and play a significant role in preventing the virus from resurging to a degree that goes beyond national and economic control.

## IMPACT OF EDUCATION AND INCOME LEVELS ON CORONAVIRUS DISEASE 2019

Certain studies looked at the potential risk of exposure to COVID-19 by stratifying data based on education and income levels [19]. These studies used occupational standing (OS), defined as the proportion of workers in each occupation with a certain level of educational qualification. Those with higher socioeconomic status (such as higher education and/or income levels) usually have access to a broad range of healthcare resources to protect themselves compared to other socioeconomic groups, which indicates the existence of health inequalities [20]. Regarding workplace exposure to COVID-19 infection, higher-education-level workers are more likely to work for employers who maintain appropriate standards of practising risk mitigation strategies, follow risk reduction measures, and provide personal protective equipment (PPE) kits to their workers [19]. Furthermore, workers with higher education levels tend to put more effort into understanding and learning about the mechanisms of COVID-19 transmission and are more willing to implement risk reduction strategies [21].

**Figure 1.**
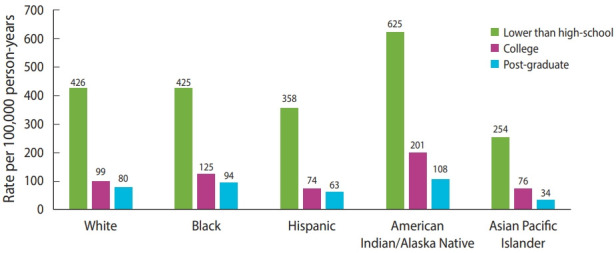
. Coronavirus disease 2019 mortality rates by race/ethnicity and education in the United States [24].

It is evident that members of certain minority racial and ethnic groups with lower education and income levels are more likely to work in occupations where social isolation is not possible [22]. Socioeconomic status, usually derived based on the income level, plays a vital role in health outcomes as those with relatively low socioeconomic status may be more likely to hold a minimum wage job or work in unsafe conditions that put them at higher risk of being infected with COVID-19 [23]. Therefore, education and income levels seem to have a strong connection with occupation conditions and disease transmission; for instance, all healthcare professionals (clinicians, pharmacists and nurses) are categorised as higher OS workers, whilst those who work in transportation, retail stores, farms, and meat processing factories are defined as lower OS workers [21]. Regardless of their race/ethnicity, individuals with an education level lower than high-school are at a higher risk of death due to COVID-19 than those with higher education levels such as college and post-graduate education (Figure 1) [24]. In terms of COVID-19 vaccination, although rapid and widespread vaccination rollouts are in place, those with lower education and income levels and those who are Black/Latino are less likely to be fully vaccinated than their counterparts [25].

## OPPORTUNITIES AND STRATEGIES FOR IMPROVEMENT

The ongoing gaps in collecting race, ethnicity, and occupation information result from a dearth of coordinated efforts, inertia, insufficient analysis of economic trends, and a lack of pioneering leadership. This leads us to the fact that the existing databases have many loopholes and valuable data are missing, or the available information is unreliable and thus not credible for analysis. In the midst of a pandemic of this scale, which seems to have vastly distributed itself disproportionately among specific racial and occupational groups, it is mandatory to have reliable systems and epidemiological databases in place that efficiently capture this essential information. This would play a central role in efficiently tracking the affected, enabling occupational epidemiologists to collaborate with the government and frame central and state-specific policies to protect the targeted groups.

Developing active surveillance systems such as occupational health surveillance is needed for a further understanding of various factors contributing to disparities in fatalities across different races, ethnicities, and occupations. Similarly, death certificates would act as an alternate approach to collect essential information related to race, ethnicity and occupation, which would make it possible to calculate more accurate mortality rates [26]. In ideal circumstances, race, ethnicity, and occupation data should be collected for all individuals who test positive for COVID-19 because one’s race and profession are indispensable determinants of health that cannot be avoided in public surveillance systems. In an attempt to overcome this barrier and facilitate the reporting of COVID-19 cases, the National Institute of Occupational Safety and Health (NIOSH) surveillance program emphasised the significance of having a systematic procedure for collection, coding, analysis, and reporting for both industry (employer’s type of business) and occupation (type of job) data during the pandemic [27]. The NIOSH has developed a system known as NIOSH Industry and Occupation Computerised Coding System (NIOCCS), which automatically codes industry and occupation information; the NIOCCS could be used to code occupation information from death certificates [28].

The Council of State and Territorial Epidemiologists Occupational Health Subcommittee’s Recommended Interim Guidance for Collecting Employment Information about COVID-19 also gives recommendations for the qualitative collection of employment information [29]. The United States utilises the Department of Commerce to maintain the standard codes for industries in North America, the North American Industry Classification system, or another method of categorisation known as the Standard Occupational Classification system, maintained by the Bureau of Labour Statistics, which can also be utilised to obtain these standard codes. Thus, establishing race, ethnicity and occupational mortality surveillance systems can help to address various health policy-related issues—not just during the current pandemic, but also in future pandemics—and potentially identify areas for intervention.

Therefore, identifying and safeguarding these highly susceptible groups is essential during this current crisis as most of these groups are involved in providing essential services to the community. The process of protecting these individuals include providing PPE and vaccines to limit their exposure, providing stable health insurance and income support, and offering sick leave (including paid leave) and compensation benefits if a worker becomes sick or shows signs and symptoms of COVID-19. In addition, such an approach may help to balance ethnic and racial disparities within the healthcare system. Although the Families First Coronavirus Act offered federally mandated paid sick leave for those unable to work due to the current pandemic and respective social distancing laws, 39% of workers who are POC are affected by exemptions in the law [30].

## CONCLUSION

Due to the high level of uncertainty posed by the pandemic, race, ethnicity, and occupation-related projections have yet to be released. It is essential to have a platform wherein race, ethnicity, and occupation data of the infected are recorded in detail, which would efficiently supplement the execution of policies that protect vulnerable professions and susceptible ethnic groups. However, there is a very scarce data pool that accurately records such information. Thus, collecting and recording this information will provide valuable insights and help public health officials identify workplace-related outbreaks and evaluate the odds of various ethnic groups and professions contracting COVID-19.

### Ethics statement

No institutional review board approval was needed since this is a perspective article.

## Figures and Tables

**Table 1.** Risk for coronavirus disease 2019 infection, hospitalization, and death by race/ethnicity in the United States compared to White population [11]

Determinants	Black or African	Hispanic or Latino	American Indian or Alaska Native
Cases (infection)	1.1 x	1.9 x	1.7 x
Hospitalization (morbidity)	2.8 x	2.8 x	3.5 x
Deaths (mortality)	2.0 x	2.3 x	2.4 x
